# Inflammatory Profiling of Schwann Cells in Contact with Growing Axons Distal to Nerve Injury

**DOI:** 10.1155/2014/691041

**Published:** 2014-04-27

**Authors:** Petr Dubový, Ilona Klusáková, Ivana Hradilová Svíženská

**Affiliations:** Department of Anatomy, Division of Neuroanatomy, Medical Faculty and Central European Institute of Technology (CEITEC), Masaryk University, 625 00 Brno, Czech Republic

## Abstract

Activated Schwann cells distal to nerve injury upregulate inflammatory mediators, including cytokines. The goal of the present study was to investigate expression of proinflammatory (IL-1**β**, TNF**α**) and anti-inflammatory cytokines (IL-4, IL-10) in activated Schwann cells in relation to growing axons distal to crush injury of rat sciatic nerves. Seven days from sciatic nerve crush, transverse cryostat sections were cut 5 mm distal to lesion and incubated for double immunostaining to indicate Schwann cells (GFAP or S100b) and individual investigated cytokines or to demonstrate growing axons (GAP43). The Schwann cells of naïve sciatic nerves and those removed from sham-operated rats displayed similar weak immunoreactivity for the investigated cytokines. In contrast, increased intensity of cytokine immunofluorescence was found in Schwann cells distal to crush lesion. The cytokine-positive Schwann cells were found in close contact with growing axons detected by immunostaining for GAP43. The results of immunohistochemical analysis distal to nerve crush injury suggest that inflammatory profiling of Schwann cells including upregulation of both pro- and anti-inflammatory cytokines does not prevent growth of axons distal to nerve crush injury.

## 1. Introduction

Inflammation is a response of the innate immune system to protect and defend the body after bacterial or viral invasions and tissue damage. The inflammatory response is orchestrated by the mobilization and interaction of several cell types and signaling molecules, thereby producing a response that is both local and systemic. Inflammatory response not only eliminates impaired cells and invading pathogens, but it also actively promotes wound healing. However, progressive and/or chronic inflammatory reaction can result in a failure to remove or resolve the initiating insult or in a dysregulated injury response that impairs tissue recovery [1]. In contrast to inflammation in other tissues, where immune cells are the main source of inflammatory mediators, the principle cells of the nervous system—neurons and activated glial cells—contribute significantly to neuroinflammatory reactions. Nervous system injuries induce upregulation of inflammatory mediators like cytokines in the neurons as well as glial cells of the central and peripheral nervous systems [2, 3]. Many studies have been directed to investigating the detrimental effects of neuroinflammation after nerve injury, including neurodegeneration or neuropathic pain induction [4, 5], but little is known about the beneficial effects of neuroinflammatory mediators.

Wallerian degeneration is a cascade of stereotypical cellular and molecular events distal to injury of nerve fibers and is considered to be a sort of innate immune reaction or neuroinflammation. The cellular events comprise inflammatory activation of Schwann cells and invasion of various types of immune cells, including macrophages, that produce cytokines and chemokines. It is well known that proinflammatory cytokines and chemokines play key roles during the early phase of Wallerian degeneration in managing the invasion of macrophages and cleaning of the myelin debris [6, 7] and that they contribute indirectly to axon regeneration. Recently, only studies directed to neuropoietic cytokines provide evidence about the direct effects of cytokines on activating the intrinsic capacity of the neurons to regenerate their axons or overcome external inhibitory cues [8].

Little is known, however, about the beneficial effects of pro- and anti-inflammatory cytokines upregulated by activated Schwann cells upon axonal growth distal to nerve injury. The goal of the present study was to investigate expression of proinflammatory (IL-1*β*, TNF*α*) and anti-inflammatory cytokines (IL-4, IL-10) in activated Schwann cells in relation to growing axons distal to crush injury of rat sciatic nerves.

## 2. Materials and Methods

### 2.1. Animals and Surgical Procedures

Eighteen pathogen-free Wistar rats (male, 250–300 g) used for the experiments were housed on a 12 h light/dark cycle at 22–24°C under specific pathogen-free conditions in the animal housing area of the Masaryk University. Sterilized standard rodent food and water were available ad libitum. All surgical procedures were carried out under deep anesthesia induced through a mixture of ketamine (40 mg/kg) and xylazine (4 mg/kg) and sterile conditions. All surgery was conducted by the same person according to protocols approved by the Animal Investigation Committee of the Faculty of Medicine, Brno, Czech Republic.

To create the nerve injury, the right sciatic nerve was exposed at the level of the midthigh by blunt dissection just proximal to its trifurcation. The nerve crush injury (*n* = 6) was carried out using a No. 5 jeweler's forceps to clamp the nerve for 30 s. Animals operated on to create nerve crush were left to survive for 7 d. Intact rats (*n* = 6) were used as naïve control, and the right sciatic nerves of sham-operated rats (*n* = 6) were exposed only without nerve injury.

### 2.2. Sections and Immunohistochemical Staining

Nerve samples were removed from rats perfused transcardially with Zamboni's fixative. The distal nerve segments were harvested 5 mm from injury point and corresponding nerve samples were also removed from the sciatic nerve of naïve and sham-operated rats.

Cryostat sections (12 *μ*m) cut transversally through naïve, sham-, and crush-operated sciatic nerves were immunostained for IL-1*β*, TNF*α*, IL-4, and IL-10 under identical conditions. A part of the nerve sections distal to lesion was double immunostained for S100b or GFAP to detect Schwann cells and GAP-43 to indicate regrowing axons. The sections were incubated with a mixture (1 : 1) of antibody against the detected molecule and antibodies for immunostaining of Schwann cells or growing axons of a different host animal (Table 1). A mixture (1 : 1) of affinity purified TRITC- or Alexa 350-conjugated donkey anti-rabbit and TRITC- or FITC-conjugated donkey anti-mouse secondary antibodies was applied with a final dilution of 1 : 100 at room temperature for 90 min. The control sections were incubated while omitting the primary antibodies. Immunostained sections were rinsed and mounted in a Vectashield aqueous mounting medium (Vector Laboratories Inc., Burlingame, CA, USA) and analyzed using a Leica DMLB epifluorescence microscope equipped with appropriate filter combinations and a Leica DFC-480 camera (Leica Microsystems Wetzlar GmbH, Germany).

## 3. Results

**Figure 1:**
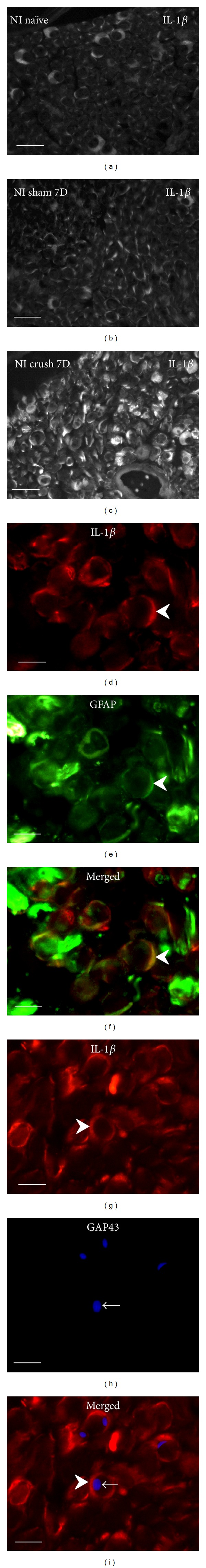
Cryostat cross sections through naïve rat sciatic nerve (a), after sham operation (b), and distal to crush for 7 d (c) immunostained for IL-1*β* revealed robust increase of immunofluorescence distal to nerve injury. Increased IL-1*β* immunofluorescence was found dominantly in Schwann cells detected by colocalization with GFAP (arrowheads, (d)–(f)). Growing axons and sprouts immunostained with GAP43 (arrows) were found in close contact with Schwann cells decorated by IL-1*β* immunostaining (arrowheads) ((g)–(i)). Scale bars for (a)–(c) = 20 *μ*m, for (d)–(i) = 5 *μ*m.

**Figure 2:**
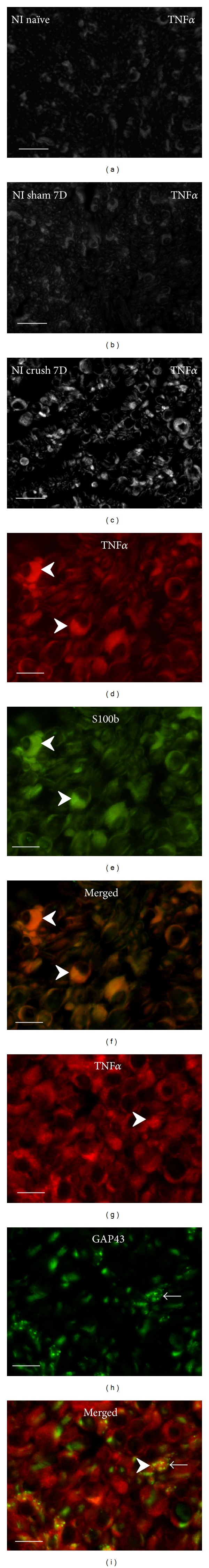
Representative cryostat sections through naïve rat sciatic nerve (a), after sham operation (b), and distal to crush for 7 d (c) immunostained for TNF*α* demonstrated strong increase of immunofluorescence intensity distal to nerve crush. Immunofluorescence staining for TNF*α* protein was observed in Schwann cells detected by colocalization with S100b immunostaining (arrowheads, (d)–(f)). Many growing axons immunostained for GAP43 (arrows) were closely related to Schwann cells decorated by TNF*α* immunostaining (arrowheads) ((g)–(i)). Scale bars for (a)–(c) = 80 *μ*m, for (d)–(i) = 10 *μ*m.

**Figure 3:**
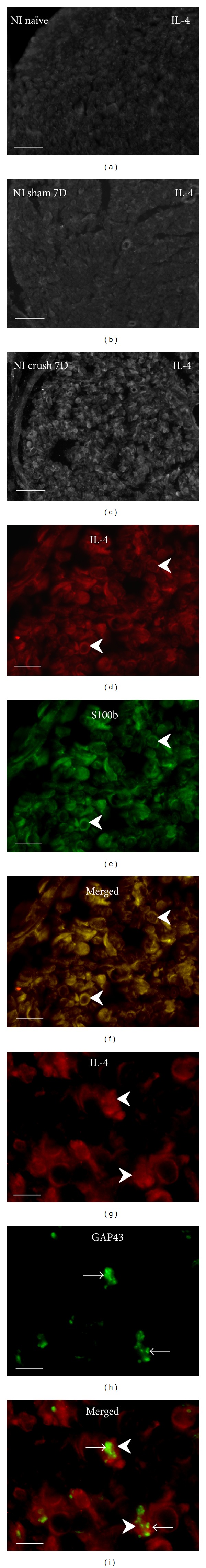
Cryostat sections through the sciatic nerve of naïve rat (a), after sham operation (b), and distal to sciatic nerve crush for 7 d (c) immunostained for IL-4. The sections of naive and sham-operated sciatic nerve displayed very weak immunofluorescence for IL-4, but moderate intensity of IL-4 immunofluorescence was observed distal to nerve crush injury. Schwann cells immunodetected by GFAP displayed also immunostaining for IL-4 at higher power magnification (arrowheads, (d)–(f)). Growing GAP43+ axons (arrows) were found in close contact with IL4+ Schwann cells (arrowheads) ((g)–(i)). Scale bars for (a)–(c) = 80 *μ*m, for (d)–(f) = 15 *μ*m, for (g)–(i) = 5 *μ*m.

**Figure 4:**
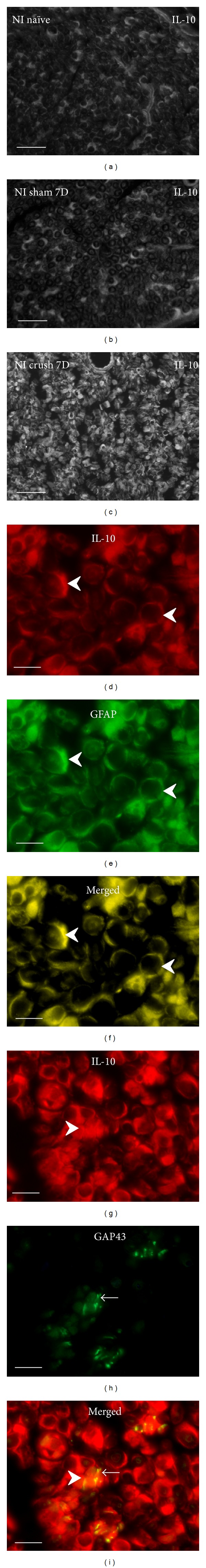
Cryostat sections through naïve rat sciatic nerve (a), after sham operation (b), and distal to crush for 7 d (c) illustrate robust increase of IL-10 immunofluorescence distal to nerve crush injury. Increased IL-10 immunofluorescence was found in GFAP+ Schwann cells (arrowheads, (d)–(f)). Many growing axons immunostained for GAP43 (arrows) were in close contact with Schwann cells immunopositive for IL-10 (arrowheads) ((g)–(i)). Scale bars for (a)–(c) = 40 *μ*m, for (d)–(i) = 10 *μ*m.

Cryostat cross sections through the sciatic nerves from naïve or sham-operated rats displayed very low intensity of immunofluorescence for IL-1*β*, TNF*α*, IL-4, and IL-10. In contrast, an increased intensity of immunostaining was detected in sections cut distal to nerve crush for 7 d. A robust increase of immunofluorescence intensity was detected for IL-1*β*, TNF*α*, and IL-10, but only moderate elevation was observed after IL-4 immunostaining when sections were incubated under the same conditions (Figures 1(a)–1(c), 2(a)–2(c), 3(a)–3(c), and 4(a)–4(c)).

In addition to activated ED-1+ macrophages and other immune cells that invaded distal nerve stumps (data not shown), immunostaining for the investigated cytokines was observed in ring-shaped cells or in patches. Double immunostaining with S100b or GFAP revealed the immunopositivity to be related to Schwann cells (Figures 1(d)–1(f), 2(d)–2(f), 3(d)–3(f), and 4(d)–4(f)). The results indicated that both proinflammatory (IL-1*β*, TNF*α*) and anti-inflammatory (IL-4, IL-10) cytokines are dominantly expressed by activated Schwann cells. The growing axons visualized by immunostaining for GAP43 were frequently observed in close contact with Schwann cells which displayed simultaneously immune reactions for IL-1*β*, TNF*α*, IL-4, or IL-10. In some cases, the GAP43+ axonal sprouts were completely enveloped by cells with immunopositivity for cytokines (Figures 1(g)–1(i), 2(g)–2(i), 3(g)–3(i), and 4(g)–4(i)).

Our results have shown that nerve crush injury for 7 d induced a higher intensity of immunostaining for proinflammatory (IL-1*β*, TNF*α*) and anti-inflammatory (IL-4, IL-10) cytokines when compared with sections through the sciatic nerves from naïve or sham-operated rats. Strong increase of immunofluorescence intensity distal to crush injury was found for IL-1*β*, TNF*α*, and IL-10 while only moderate intensity was observed for IL-4 immunoreaction. Double immunostaining revealed that both proinflammatory and anti-inflammatory cytokines were produced not only by resident and recruited immune cells but also by Schwann cells 5 mm distal to nerve crush injury.

## 4. Discussion

It is well known that synthesis of proinflammatory and anti-inflammatory cytokines is increased distal to nerve injury [9, 10]. Most cytokines are upregulated in the distal stump of injured nerve in two or three waves. The upregulation of cytokines by activated Schwann cells during the early phase of Wallerian degeneration (1–3 days after nerve crush) is implicated predominantly in myelin destruction and recruitment of hematogenous macrophages [6, 7, 11]. This early peak is followed by a second, later phase of increased cytokine expression [9, 10]. A second phase of IL-1*β*, TNF*α* and IL-10 upregulation [12, 13] and period of axon growth [14] occur within 7 days after nerve crush injury. Our immunofluorescence staining results have shown that, in addition to invaded macrophages and other immune cells [15–17], Schwann cells constitute another robust source of both pro- and anti-inflammatory cytokines distal to nerve crush injury for 7 d. In addition, double immunostaining revealed that GAP43+ growing axons are in close contact with Schwann cells strongly decorated by immunofluorescence for IL-1*β*, TNF*α*, IL-4, and IL-10 (Figures 1(d)–1(i), 2(d)–2(i), 3(d)–3(i), and 4(d)–4(i)). Tissue and peripheral nerve injury lead to systemic and local inflammatory reaction that is accompanied by elevated levels of mediators, including proinflammatory cytokines like IL-1*β*, IL-6, and TNF*α* [18, 19]. However, sections of the sciatic nerves from naïve and sham-operated rats incubated under the same condition displayed very similar intensity of immunofluorescence for the investigated cytokines (Figures 1(a), 1(b), 2(a), 2(b), 3(a), 3(b), and 4(a), 4(b)). This indicates no significant effect of surgical treatment (skin incision, tear of muscle) on local expression of cytokines in the sciatic nerve of our experimental animals.

Published results regarding cytokine effects upon axon regeneration are frequently controversial, because cytokines, like inflammation itself, constitute a so-called “double-edged sword” with respect to nerve regeneration. These opposing effects are related to the degree and timing of inflammatory reactions in severed nerve. A beneficial effect of local inflammation upon axon regeneration probably depends upon orchestrated production and control of cytokine levels. Our results of double immunostaining for cytokines and GAP43 indicate that inflammatory profiling of Schwann cells, expressed by upregulation of proinflammatory and anti-inflammatory cytokines, does not prevent growth of axons distal to nerve crush injury. This suggests that proinflammatory cytokines have a role not only during the early phase of Wallerian degeneration but also at later periods of time after nerve lesion when axons regenerate. A beneficial effect of local upregulation of proinflammatory cytokines distal to nerve injury upon axon regeneration may occur either in synergy with neurotrophins [20] or, if their detrimental effects are overcome, by axon promoting agents. In addition, this may depend upon orchestrated production of anti-inflammatory cytokines (IL-4, IL-10) that are able to control levels of proinflammatory cytokines. Chronic overproduction of cytokines after traumatic nerve injury or other nerve diseases has been implicated in conditions inducing neuropathic pain [21]. However, optimal levels of cytokines and the mechanisms of their balance to achieve beneficial effects in lesioned peripheral nerve are still unknown.

Although proinflammatory cytokines are mostly considered to be molecules with detrimental effect on axon growth, some* in vitro* and* in vivo* results have been accumulated to support the implication of individual cytokines in promoting axon growth after nerve injury. Increased expression of IL-1*β* is present in the immature Schwann cells of the distal nerve stump in the early stage of Wallerian degeneration, and this disappears thereafter when Schwann cells begin their remyelination [10]. IL-1*β* regulates synthesis of nerve growth factor (NGF) by Schwann cells and fibroblasts [22, 23] and, together with neurotrophin-3, it synergistically promotes neurite growth [24]. Moreover, this cytokine is able to overcome MAG-induced RhoA activation and axon growth inhibition to promote sensory axon outgrowth under* in vitro* and* in vivo* conditions [25, 26].

Controversial results also have been published about direct effects of TNF*α* upon axon growth during peripheral nerve regeneration. While TNF*α* may promote motor functional recovery after crushing of peripheral nerve [27], it also has been found that systemic and local administration of the TNF*α* antagonist etanercept enhanced the rate of axonal regeneration [28]. While there are also results showing that TNF*α* reduces neurite sprouting [29], a recent paper by Saleh and coworkers [30] proved an increased branching of primary sensory neurons after application of TNF*α* through an NF-*κ*B-dependent pathway.

In summary, there is a growing body of evidence that IL-1*β* and TNF*α* in optimal concentrations are important for stimulation of axonal growth and recovery of functional innervation after nerve injury and that neutralization of these proinflammatory cytokines most likely impairs peripheral nerve regeneration [12].

IL-4 and IL-10 are prototypical anti-inflammatory cytokines that modulate expression of proinflammatory cytokines and have pivotal importance in axon plasticity and outgrowth [31]. IL-4 modulates macrophage activity through globally suppressing proinflammatory cytokines, but nothing is known about its upregulation in activated Schwann cells distal to nerve injury. It has been demonstrated by* in vitro* culture experiments that axonal growth of the primary sensory neurons can be stimulated by IL-4 in combination with NT-4 [20]. This suggests that IL-4 produced by immune and Schwann cells may stimulate axonal growth in synergy with local secretion of neurotrophins. Expression of IL-10 after nerve injury is an interesting subject, because it attenuates production of proinflammatory cytokines IL-1*β* and TNF*α*. Significant increase of IL-10 and IL-10-immunopositive cells has been observed 7 d after nerve injury [13, 32]. Thus, IL-10 may promote axonal regeneration by inhibiting overproduction of proinflammatory cytokines.

## 5. Conclusions

In contrast to intact nerve, Schwann cells distal to crushed sciatic nerve displayed increased immunostaining for proinflammatory (IL-1*β*, TNF*α*) and anti-inflammatory (IL-4, IL-10) cytokines 7 d after nerve lesion. The cytokine-positive Schwann cells were in close contact with growing GAP43-positive axons. The results suggest that a concomitant induction of proinflammatory and anti-inflammatory cytokines may maintain a balance in the inflammatory reaction of Schwann cells and their involvement in promoting axonal growth.

## Figures and Tables

**Table 1:** Primary antibodies used for immunofluorescence detection of cytokines, Schwann cells, and growing axons.

	Antibody	Source	Product	Dilution
IL-1*β*	mAb	Mouse	Serotec	1 : 100
IL-4	pAb	Rabbit	Santa Cruz	1 : 100
IL-10	pAb	Rabbit	Serotec	1 : 500
TNF*α*	pAb	Rabbit	Abcam	1 : 1000
S100	mAb	Mouse	Sigma	1 : 500
GFAP	pAb	Rabbit	Dako	1 : 250
GAP43	mAb	Mouse	Abcam	1 : 500
GAP43	pAb	Rabbit	MyBioSource	1 : 500

Monoclonal (mAb), polyclonal (pAb) antibody.
